# Axonal Transport and Mitochondrial Function in Neurons

**DOI:** 10.3389/fncel.2019.00373

**Published:** 2019-08-09

**Authors:** Amrita Mandal, Catherine M. Drerup

**Affiliations:** Unit on Neuronal Cell Biology, Eunice Kennedy Shriver National Institute of Child Health and Human Development, National Institutes of Health, Bethesda, MD, United States

**Keywords:** mitochondria, axonal transport, dynein, kinesin, mitochondrial dynamics, neurodegenerative disease

## Abstract

The complex and elaborate architecture of a neuron poses a great challenge to the cellular machinery which localizes proteins and organelles, such as mitochondria, to necessary locations. Proper mitochondrial localization in neurons is particularly important as this organelle provides energy and metabolites essential to form and maintain functional neural connections. Consequently, maintenance of a healthy pool of mitochondria and removal of damaged organelles are essential for neuronal homeostasis. Long distance transport of the organelle itself as well as components necessary for maintaining mitochondria in distal compartments are important for a constant supply of healthy mitochondria at the right time and place. Accordingly, many neurodegenerative diseases have been associated with mitochondrial abnormalities. Here, we review our current understanding on transport-dependent mechanisms that regulate mitochondrial replenishment. We focus on axonal transport and import of mRNAs and proteins destined for mitochondria as well as mitochondrial fusion and fission to maintain mitochondrial homeostasis in distal compartments of the neuron.

## Introduction

Neurons have one of the largest and most complex architectures of all cells in the human body. The peripheral arbors of a neuron can extend for extraordinary distances. In the substantia nigra, for example, the sum total of the axonal arbor may span up to ∼4.5 m (Bolam and Pissadaki, 2012). In order to form and maintain such an enormous structure, active transport of mRNAs, proteins, and organelles throughout the cell is essential. One organelle of critical importance is mitochondria, as they play a central role in ATP generation, metabolite synthesis and calcium buffering among other lesser known functions (Werth and Thayer, 1994; Misgeld and Schwarz, 2017). The importance of mitochondria in neuronal health is exemplified by neurodegenerative diseases such as Alzheimer’s Disease, Amyotrophic Lateral Sclerosis (ALS), and Parkinson’s disease among others (Table 1). In these diseases, pathology often correlates with defects in mitochondrial localization or function (Rui et al., 2006; De Vos et al., 2007; Chen and Chan, 2009; Schon and Przedborski, 2011; Wang et al., 2011; Reddy et al., 2012) leading many to postulate that the proper distribution and maintenance of a healthy pool of mitochondria is essential for the health of a neuron.

**TABLE 1:** Neurodegenerative diseases with mitochondrial abnormalities.

**Disease**	**Clinical phenotypes**	**Gene (s)**	**Mitochondrial phenotypes**	**References**
Alzheimer’s disease	Progressive cognitive decline	*APP*, *Tau, Presenilin-1, Presenilin-2*	Oxidative stress, mitochondrial dysfunction, reduced mitochondrial motility	Pereira et al., 1998; Canevari et al., 1999; Reddy et al., 2004
Amyotrophic lateral sclerosis	Muscle weakness and progressive paralysis	*SOD1*, *TDP4*, *VAPB*	Defective Ca^2+^ buffering, increased Complex I activity, mitochondrial transport arrest	Bowling et al., 1993; Fujita et al., 1996; De Vos et al., 2007
Parkinson’s disease	Tremor and involuntary movements	*Pink1*, *Park2, SNCA*,	Complex I deficiency, inhibition of mitochondrial motility, failed mitophagy	Bindoff et al., 1989; Parker et al., 1989; Schapira et al., 1989; Wang et al., 2011
Charcot-Marie-Tooth	Weakness largely in the lower extremities	*Mfn*	Impaired mitochondrial fusion, arrested mitochondrial mobility	Kijima et al., 2005; Pedrola et al., 2005
Huntington’s disease	Involuntary and uncoordinated movement	*Htt*	Reduced Complex I activity and membrane potential, impaired mitochondrial trafficking	Parker et al., 1990; Arenas et al., 1998; Chang et al., 2006
Optic atrophy	Visual dysfunction	*Opa1*	Impaired mitochondrial fusion	Zanna et al., 2008
Spastic paraplegia	Spasticity and weakness of the lower limbs	*Paraplegin, HSP60*	Oxidative phosphorylation dysfunction	Casari et al., 1998

## Mitochondrial Transport and Anchoring Machineries

Mitochondrial distribution throughout the neuron is coordinated by microtubule-based transport machinery. This includes an array of motor proteins and adaptors to move  these  organelles  along

the microtubule tracks. In the axon, anterograde transport (microtubule plus end directed, toward the axon terminal) of mitochondria primarily utilizes the Kinesin-1 motor (Pilling et al., 2006). The attachment of Kinesin-1 to mitochondria is mediated by the membrane anchor proteins RhoT (a small Rho GTPase) and its motor adaptors Trak1 and Trak2. Originally discovered in *Drosophila*, these proteins are essential for microtubule based transport of mitochondria into the dendrite and axon (Stowers et al., 2002; Glater et al., 2006; Russo et al., 2009).

Retrograde transport (microtubule minus-end directed in axon, toward the cell body) employs a single motor protein complex, Cytoplasmic dynein (hereafter refered to as dynein; Schnapp and Reese, 1989). The detailed mechanism of how the dynein motor binds to mitochondria for retrograde transport is still largely unknown. Trak2 appears to participate in this process as disruption of Trak2 in hippocampal neurons resulted in a significant decrease in the percentage of dynein-mediated mitochondrial movement into dendrites (Loss and Stephenson, 2017). Additionally, recent work from our lab has established Actr10 (Arp11/Arp10p) as an important mediator for retrograde mitochondrial transport. Loss of Actr10 leads to mitochondrial accumulation in axon terminals due to selective impairment of retrograde transport. Meanwhile, localization and transport of other dynein cargos, such as lysosomes and peroxisomes, are not disrupted (Drerup et al., 2017). A complete and detailed understanding of the mechanistic regulation of both anterograde and retrograde mitochondrial motility are still lacking and, thus, warrant further examination.

During development, mitochondrial transport in the anterograde and retrograde directions is frequent, with few mitochondria pausing for extended periods of time in the axon in particular. However, as the neuron ages, mitochondrial transport becomes less frequent (Morsci et al., 2016). Real time imaging of mitochondrial movement in axons of cultured neurons at 28 days *in vitro* suggests that 95% of mitochondria are stationery at this point over a period of 30 min of imaging (Lewis et al., 2016). Studies done *in vivo* in exposed mouse sciatic nerve have shown approximately 22% of mitochondria are motile in adult animals at 45 days of age (Magrane et al., 2014). Studies in zebrafish axons at various developmental time-points match more closely with that seen in mouse sciatic nerve imaging, with approximately 50% of mitochondria motile at 5 days post-fertilization (Mandal et al., 2018). These studies, however, have been done over a period of minutes. The temporal dynamics of mitochondrial transport and docking over longer periods of time are largely unknown.

Movement of mitochondria needs to be coupled with docking of the organelle for proper distribution throughout the neuron. This docking is regulated by local environmental cues and the use of docking proteins which anchor mitochondria at specific locations (Cai and Sheng, 2009). Stationary mitochondria are often found in areas of high ATP demand (Spillane et al., 2013). This local ATP source is important for axonal branching, local protein translation, sodium/potassium pump activity necessary for maintaining the neuron’s polarization, and synaptic transmission (Erecinska and Dagani, 1990; Spillane et al., 2013; Cioni et al., 2019; Rangaraju et al., 2019). For example, a previous study found that in the presence of nerve growth factor (NGF) axonal branching occurs at a site of stalled mitochondria. Inhibition of respiration prevented the branching, indicating that ATP produced from the stalled mitochondria is important for this phenomenon (Spillane et al., 2013). Additionally, mitochondria function to buffer calcium in neurons. Stationary mitochondria at synapses can act as a buffering chamber to take up excess cytosolic calcium, which is necessary to regulate calcium-dependent synaptic activity (Yi et al., 2004; Zhang et al., 2010). Mitochondrial anchoring at areas of high calcium, like the synapse, is accomplished by the ion itself. High levels of calcium effect the conformation of the EF-hand structural domain on RhoT. This modulates the affinity of the anterograde motor kinesin for microtubules, regulating anterograde movement. Interestingly, elevation of calcium stops all mitochondrial movement, implicating this ion in the regulation of retrograde movement as well (Saotome et al., 2008). This effect could also be through RhoT or another calcium sensitive protein.

Mitochondrial docking is facilitated by anchoring proteins such as the neuron-specific protein Syntaphilin (Snph). Snph acts on the mitochondrial outer membrane (OM) and keeps the organelle stationary by bridging it with the microtubule cytoskeleton. Mice lacking Snph show a significant increase in the motile mitochondrial population (Kang et al., 2008). This interaction appears to also require members of the retrograde transport apparatus as the dynein light chain LC8 can facilitate the Snph–microtubule interaction to immobilize mitochondria (Chen et al., 2009).

Mutations in several motor proteins important for mitochondrial transport have been associated with neurodegenerative diseases. A missense mutation in the N-terminal motor domain of the Kinesin-1 (Conventional Kinesin) was found in patients with hereditary spastic paraplegia (HSP), an axonal degeneration disorder of motor and sensory neurons (Reid et al., 2002). Additionally, Kinesin-1 mutations have been associated with ALS in patient populations. Recent genome wide analysis and exome sequencing have found mutations in the C-terminal cargo binding domain of *Kif5A* (Kinesin-1 isoform) in ALS patients (Brenner et al., 2018; Nicolas et al., 2018). Loss of function mutation in KIF1Bβ (Kinesin-3 motor) is associated with Charcot-Marie-Tooth Disease Type 2A (CMT2A), a peripheral neuropathy with progressive loss of muscle function (Zhao et al., 2001). Additionally, mutations is motor associated proteins have been associated with disease states. Mutation of the mitochondrial fusion protein 2 (*Mfn2*) gene is one of the most common causes of CMT2A. Expression of disease-associated *Mfn2* mutations *in vitro* results in severe mitochondrial transport defects (Baloh et al., 2007). Mutations in *p150*^(glued)^, a subunit of the dynein activator dynactin, have been associated with motor neuron degeneration and ALS (Levy et al., 2006). Despite the correlative evidence pointing to a relationship between transport of mitochondria and disease, a causative link between the two is still lacking.

Mitochondrial transport is well-studied in the field of mitochondrial biology and has been elegantly reviewed previously (MacAskill and Kittler, 2010; Saxton and Hollenbeck, 2012; Sheng and Cai, 2012; Schwarz, 2013; Maday et al., 2014; Misgeld and Schwarz, 2017). Instead of focusing on a well-covered topic, we will review recent developments in two mechanisms important for maintenance of this organelle in the neuron which require axonal transport: replenishment of mitochondrial proteins through active transport of proteins and their precursor mRNAs and mitochondrial dynamics. We will discuss in detail the role of mitochondria in axonal protein synthesis and mitochondrial protein import machinery as well as the role of mitochondrial dynamics in mitochondrial maintenance.

## Local Mitochondrial Protein Synthesis: Mitochondria as a Fuel Source and Platform for Local Translation

Since the somatodendritic compartment contains the majority of ribosomes and the nucleus, for many years it was an attractive hypothesis that protein synthesis occurs exclusively in or near the cell body, with proteins transported to their functional target. This necessitates an efficient mechanism for bringing these proteins to the organelle no matter where it is. Although significant protein synthesis does happen in the soma, relying solely on their long-distance transport is energy expensive for the complicated and extended geometry of neurons and limits the speed with which cells can react to local cues. Research in the past 20–30 years has shown the presence of local translation far from the neuronal cell body in the distal dendrite and axon and, specifically, in pre- and post-synaptic terminals. Local axonal translation is now known to be critical for axonal guidance, synaptic plasticity, growth cone formation, axon branching and maintenance of mitochondrial membrane potential (Campbell and Holt, 2001; Verma et al., 2005; Aschrafi et al., 2008; Natera-Naranjo et al., 2012; Younts et al., 2016; Wong et al., 2017). In addition, it is thought that local translation provides the flexibility needed in remote subcellular compartments to modulate their proteome in order to keep up with local demand and external cues.

Local translation of proteins is likely especially important for mitochondria. This organelle relies on its own mitochondrial DNA (mtDNA) as well as nuclear DNA for proteins that are essential for its function and maintenance. The majority of mitochondrial proteins (∼99%) are nuclear encoded and only ∼1% of them are transcribed from a relatively small, 16.6 Kb circular mtDNA genome in humans (Taanman, 1999). Interestingly, this mtDNA specifically encodes thirteen hydrophobic inner membrane proteins which are all important components of the oxidative phosphorylation system (Pagliarini et al., 2008). Thus, any mutation in the mitochondrial genome would significantly affect ATP production. Recent data suggests crosstalk exists between the nuclear and mitochondrial translation machinery. For example, translation defects in nuclear encoded Cytochrome C oxidase, an essential electron transport chain protein, leads to a halt in translation of mtDNA encoded Cox1 in mitochondria. This is likely through a direct interaction between the RNA and protein in the organelle (Richter-Dennerlein et al., 2016). An important and understudied question is how mitochondrial proteins derived from the nuclear genome arrive at the organelle. It is likely that active mRNA transport and protein transport to distal sites accomplishes at least a portion of mitochondrial protein renewal.

**FIGURE 1:**
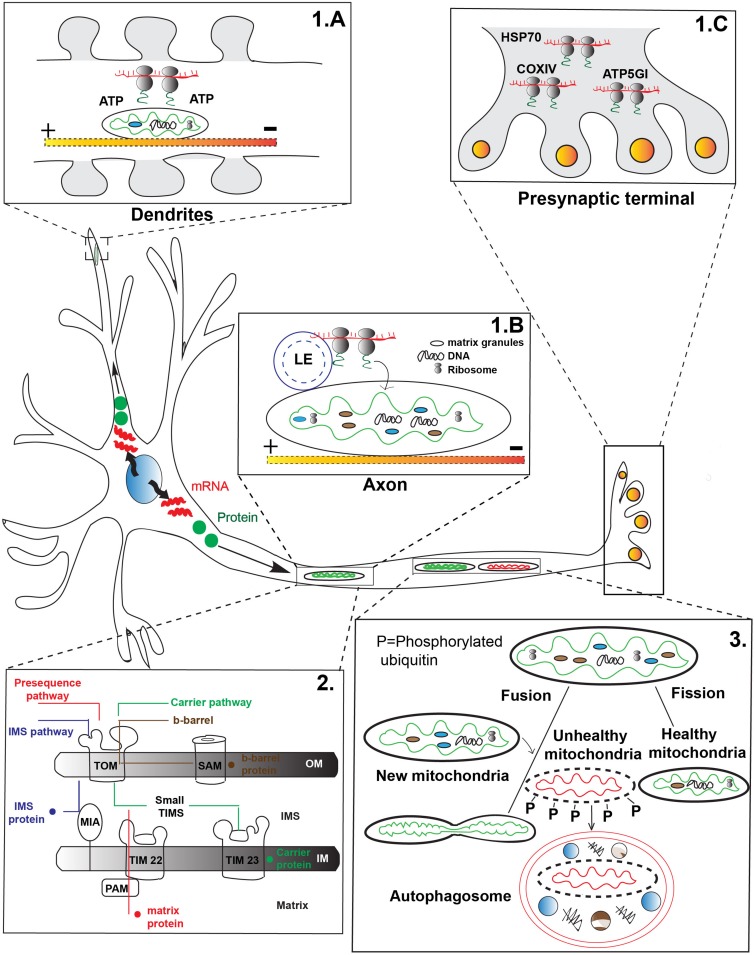
Transport-dependent mechanisms of mitochondrial maintenance. **(1)** mRNAs are transported to various regions of the neuron for local translation of mitochondrial proteins. **(A)** mRNA transported to mitochondria can be translated for local use, such as in dendritic spines during synaptic remodeling. Mitochondria are thought to generate energy for local translation. **(B)** Local translation of mitochondrial proteins from mRNA transported on late endosomes (LE) that pause on a mitochondria in axons has been demonstrated. **(C)** Polyribosomes containing mRNAs important for mitochondrial function are found in the pre-synaptic terminal of photoreceptors, indicating active transport of the mRNAs to this site. **(2)** After either local protein synthesis from transported mRNAs or protein transport to mitochondria, proteins must be imported into the organelle. Four major types of mitochondrial protein import exist: Pre-sequence pathway (red) primarily for matrix proteins; Intermembrane space (IMS) protein transport pathway (blue) important for cysteine-rich IMS proteins; Carrier protein pathway (green) for transmembrane proteins in the inner membrane; and the Outer membrane (OM) β-barrel protein import pathway (brown) for transmembrane proteins destined for the OM. TOM, translocase of the outer membrane; TIM, translocase of the inner membrane; SAM, sorting and assembly machinery; PAM, pre-sequence translocase associated motor; MIA, mitochondrial intermembrane space import and assembly machinery; OM, Outer membrane; IM, Inner membrane; IMS, Inner membrane space. Yellow-red shaded line indicates microtubule in panels **A,B**. **(3)** Mitochondria undergo continuous cycles of fusion and fission to help replenish the organelle. Fusion with younger mitochondria (green) which move in the anterograde direction from the cell body is thought to replenish proteins and lipids important for mitochondrial survival. Mitochondrial fission has been postulated to remove damaged mitochondrial components for degradation (red). Mitochondria are targeted for mitophagy after fission through phosphorylation (P) dependent events.

One mechanism of mitochondrial protein replenishment involves the active transport of mRNAs in RNA granules to mitochondria for local translation. Global gene expression analysis revealed more than one hundred nuclear encoded mitochondrial mRNAs are enriched in axons and pre-synaptic nerve terminals (Aschrafi et al., 2016). Heterogeneous populations of transcripts and polyribosomes including the mRNA for nuclear encoded mitochondrial chaperone HSP70 have been found in the pre-synaptic nerve terminals of photoreceptor neurons (Figure 1C). Some of these mRNAs, such as COXIV, ATP5GI, and ATP synthase, are critical for mitochondrial function and neuronal survival (Aschrafi et al., 2016). Furthermore, there is evidence of local translation in direct association with mitochondria. Studies in yeast have shown that nuclear encoded mRNA as well as cytoplasmic ribosomes associate with the mitochondrial OM. This suggests the local translation apparatus is present in the vicinity of the organelle (Corral-Debrinski et al., 2000). Using high resolution microscopy, Cioni et al. (2019) have recently shown strong co-localization among RNA granules, mitochondria, and late endosomes in cultured mammalian neurons. Furthermore, this study has also found mitochondrial proteins are synthesized on the late endosomes which share close physical proximity to mitochondria (Cioni et al., 2019). This data supports local translation of mitochondrial proteins on the surface of the organelle which could support the maintenance of the organelle.

Proper intracellular transport of the newly transcribed mRNA and the ribosome machinery is essential for local translation. RNA binding proteins help to guide the newly transcribed mRNA to its local translation site in axons and dendrites. Nascent mRNA associate with the RNA binding proteins to form RNA granules. Subsequently the RNA granule is transported along the microtubule track by motor proteins to reach their destined subcellular compartment. In yeast, the RNA binding protein Puf3 was found to interact selectively with nuclear encoded mRNAs for mitochondrial proteins that localize to mitochondria for translation (Saint-Georges et al., 2008). In mammalian cell culture the RNA binding protein, Splicing Factor Proline and glutamine rich (SFPQ) colocalizes with ribosomes in close proximity to mitochondria. One of the target RNAs for SFPQ is Lamin B2, local translation of which is critical for mitochondria. Inhibition of local axonal synthesis of Lamin B2 leads to mitochondrial dysfunction followed by axon degeneration (Yoon et al., 2012; Cosker et al., 2016).

Together, the current evidence supports mRNA transport and local translation of at least a subset of mitochondrial proteins in distal regions of the neuron. With the advent of sensitive techniques to study cell type specific local translation *in vivo* the precise function of this process in the biogenesis and maintenance of existing organelles promises to provide exciting advances to our understanding of mitochondrial biology in neurons.

While evidence exists for the local translation of mitochondrial proteins, whether this organelle plays a passive or active role in local protein synthesis is a current topic of investigation. One potential role of mitochondria in local protein synthesis, for mitochondrial and potentially non-mitochondrial proteins, is to act as a fuel source. A recent study by Rangaraju et al. (2019) demonstrated that docked mitochondria act as a local fuel source for translation at synapses in spatially confined dendritic pockets (Figure 1A). Inhibition of this regional power source by overexpressing mitochondrial fission factor (MFF; enhances mitochondrial fission) perturbs local translation. This data suggests that both mitochondrial form and function are under tight regulation which is necessary for local protein synthesis at the synapse (Rangaraju et al., 2019).

Mitochondria may also serve as a platform for local translation (Figure 1B). Cioni et al. (2019), recently provided evidence that RNA granules trafficked with late endosomes often stall on mitochondria for local protein synthesis. Additionally, they were able to show that this local synthesis is critical for mitochondrial function: expression of mutant forms of the late endosome protein *Rab7a*, which are responsible for Charcot-Marie-Tooth Disease type 2B, caused compromised axonal protein synthesis on mitochondria, impairment of axonal viability along with dysmorphic mitochondria and altered trafficking of the organelle. This study suggests mitochondria can serve as a ‘translational hotspot’ for protein synthesis important for the neuronal integrity and mitochondrial function (Cioni et al., 2019).

## Protein Import Machinery

Mitochondrial proteins encoded by the nuclear genome which are synthesized in the cytoplasm must be imported into the organelle. This is regulated, at least partially, by a targeting signal in the pre-protein sequence which directs them to the correct subcompartment. There are four well-defined major categories of protein import pathways for different subcompartments of mitochondria (Figure 1, panel 2). The classical protein import mechanism is the Pre-sequence pathway (Kunkele et al., 1998; Neupert and Herrmann, 2007). Most matrix targeted and inner membrane proteins are imported via this route. The protein precursors (pre-proteins) carry a N-terminal positively charged amphipathic helix sequence which are recognized by the surface receptor TOM20 of the OM. Next the cleavable precursor protein moves to the inner membrane translocase TIM23 (Brix et al., 1997; van Wilpe et al., 1999; Abe et al., 2000; Meisinger et al., 2001; Saitoh et al., 2007). The negative membrane potential across the inner membrane helps generate the electromotive force required to carry the positively charged pre-protein to the TIM23 (Martin et al., 1991). The ATP driven pre-sequence translocase associated motor (PAM) along with the heat shock protein 70 (mtHsp70) then helps transfer the protein into the mitochondrial matrix (Chacinska et al., 2005; Mapa et al., 2010). The pre-proteins are proteolytically cleaved inside the matrix by the mitochondrial processing peptidase (MPP). Alternatively, pre-proteins destined to the inner mitochondrial membrane enter via two routes. In the ‘stop transfer’ pathway a hydrophobic segment after the pre-sequence arrests the protein at the translocase TIM23 followed by lateral insertion in to the inner membrane (Glick et al., 1992; Meier et al., 2005). In the conservative pathway, proteins are partially or completely transferred to the matrix then inserted back to the inner membrane via the oxidase assembly translocase machinery which is conserved across species (He and Fox, 1997; Hell et al., 1998).

The hydrophobic inner membrane proteins such as the ADP/ATP carriers or phosphate carriers are transferred by the Carrier pathway. Rather than the N-terminal pre-sequence they have internal target sequence. These proteins use the TOM70 surface receptor to pass the OM. Next, they bind with the small TIM chaperons in the inner membrane space followed by insertion in the inner membrane by the carrier translocase TIM22. Insertion to the inner membrane is driven by the membrane potential (Endres et al., 1999; Curran et al., 2002; Rehling et al., 2003).

The third type of precursor proteins are cysteine rich intermembrane space (IMS) proteins which are imported by the mitochondrial IMS import and mitochondrial intermembrane space assembly (MIA) machinery. The MIA40 channel in the inner membrane catalyzes the formation of an intramolecular disulfide bond with the incoming pre-protein which is essential for substrate release in the IMS, stable folding and prevention of reshuffling of the protein back to the cytoplasm (Chacinska et al., 2004; Naoe et al., 2004; Banci et al., 2009).

Finally, there are two types of mitochondrial OM proteins which are imported in specific manners: β-barrel and α-helical OM proteins. The β-barrel proteins are transferred to the OM by the standard TOM translocase and small TIM chaperones. Following transfer, the sorting and assembly machinery (SAM) facilitates insertion to the OM. The detailed molecular mechanism of α-helical protein import is not fully understood yet, but previous work suggests at least some α-helical proteins skip the classic TOM translocase and utilize some yet undefined route to the mitochondrial OM (not depicted in Figure 1; Paschen et al., 2003; Wiedemann et al., 2003; Kutik et al., 2008).

Evidence for how proteins arrive at mitochondria prior to import in distal neuronal compartments is still largely lacking as it has not been a focus of active investigation. Therefore, we do not know the degree to which local protein import into mitochondria actually occurs in axons or dendrites. Given the relationship between proteome stability and mitochondrial health, this is an area that deserves active investigation.

## Protein Import, Dynamics and Quality Control

Defective protein import in general is, perhaps not surprisingly, detrimental to mitochondrial and organismal health. Mitophagy, i.e., selective degradation of damaged mitochondria via autophagy, is important for mitochondrial quality control. Protein import and mitophagy are inextricably linked processes in cells as import of Pink1 plays a key role in Pink1/Parkin mediated mitophagy. In a healthy mitochondria Pink1 is partially imported to the inner membrane via the TOM and the TIM23 translocases. Next the transmembrane domain of Pink1 is cleaved by the inner membrane rhomboid protease PAR which destabilizes Pink1. In a depolarized mitochondrion, due to the loss of membrane potential, Pink1 is not internalized and it accumulates on the OM and recruits the ubiquitin ligase Parkin. Parkin and accessory factors including p62 phosphorylate and ubiquitinate several mitochondrial proteins which marks the damaged mitochondria to be engulfed by an autophagosome for retrograde transport and degradation (Clark et al., 2006; Jin et al., 2010; Narendra and Youle, 2011; Lazarou et al., 2012; Yamano and Youle, 2013). Homozygous or compound heterozygous mutations in Pink/Parkin are associated with autosomal recessive early onset Parkinson’s disease (Narendra and Youle, 2011; Corti and Brice, 2013; Pickrell and Youle, 2015). Pink1 deficient mice show age dependent protein import defects in non-neuronal cells (Gispert et al., 2009). What is still not known is how much mitophagy happens in a neuron in non-pathological situations. Interestingly, Pink1 and Parkin deficient mice do not have any neurodegeneration phenotype. Thus, it is possible other quality control pathways are at play to maintain neuronal mitostasis such as mitochondrial proteases, mitochondria-derived vesicles, and macroautophagy.

The inner membrane fusion protein Opa1 is an additional example of how mitochondrial protein import is intricately related to mitochondrial dynamics and quality control of the organelle. In yeast, after successful import, Opa1 exists as a short isoform in the IMS as well as a long isoform inserted in the inner membrane. The proper balance between the two isoforms is important for mitochondrial fusion. ATP driven motor function is necessary for the formation of the two isoforms of Opa1 in yeast and mammals (Song et al., 2007; Ehses et al., 2009). Mutations in both the motor and *opa1* have been implicated in neurodegenerative disorders (Zanna et al., 2008; Quiros et al., 2012). More work is needed to understand the complexity and interdependence of protein import with other mitochondrial properties.

## Mitochondrial Dynamics and Neuronal Health

Mitochondrial fusion and fission are mediated by dynamin-related GTPases which are conserved across eukaryotes, from yeast to humans (Figure 1, panel 3). Fusion is an important stress response pathway in which mitochondria can exchange material. This is thought to complement mitochondrial protein and lipid content in order to overcome detrimental effects of environmental or metabolic stress (Tondera et al., 2009; Gomes et al., 2011; Rambold et al., 2011). OM fusion is mediated by Mitofusin 1 (Mfn1) and Mitofusin 2 (Mfn2) while the inner membrane fusion utilizes Opa1, among other proteins (Hermann et al., 1998; Rapaport et al., 1998; Wong et al., 2000, 2003; Santel and Fuller, 2001; Eura et al., 2003; Santel et al., 2003; Sesaki et al., 2003; Friedman and Nunnari, 2014). Mitochondrial OM fusion is almost always accompanied by the inner membrane fusion; although, in cases of loss of inner membrane potential or mutation of *opa1*, OM fusion still occurs without corresponding inner membrane fusion (Olichon et al., 2003).

Fission utilizes the cytosolic dynamin-like GTPase Drp1. Drp1 is recruited to the mitochondrial OM by the receptors Fis1 and Mff, most often at endoplasmic reticulum contact sites. Next, Drp1 oligomerizes in a GTP hydrolysis dependent manner which leads to mitochondrial division (Friedman et al., 2011; Mears et al., 2011; Friedman and Nunnari, 2014). A number of other Drp1 receptors on mitochondrial OM such as Mid49, Mid51, and GDAP1 have also been reported, suggesting there are multiple ways to regulate fission (Loson et al., 2013). Mitochondrial fission can be regulated by different protein modifications, most importantly phosphorylation of Drp1 at selected serine residues. For example, phosphorylation at the S616 site activates fission while phosphorylation at S637 impairs the GTPase activity and fission (Chang and Blackstone, 2007; Cribbs and Strack, 2007; Taguchi et al., 2007). Calcium signaling can also affect mitochondrial dynamics by regulating Drp1 phosphorylation. Calcium influx via the voltage dependent calcium channel promotes phosphorylation at the S600 site of Drp1 via activation of calcium/calmodulin dependent protein kinase Iα (Han et al., 2008). This phosphorylation leads to Drp1 recruitment to mitochondria followed by fission. Additionally, hyperglycemia induced mitochondrial fission has been found to be mediated by Drp1 phosphorylation at S600 residue via Rho-associated coiled coil-containing protein kinase 1 (ROCK1; Wang et al., 2012). Other important post-translational modifications of Drp1 includes glycosylation, sumoylation, S-nitrosylation and ubiquitination (Chang and Blackstone, 2010; Otera et al., 2013). Together, this data points to Drp1 as nexus for regulating mitochondrial form and function in response to cellular and extracellular cues.

The fusion/fission genes are important for an organism’s survival as knockout mice for (*Mfn1*, *Mfn2*, and *Drp1*) are embryonic lethal (Chen et al., 2003; Ishihara et al., 2009; Wakabayashi et al., 2009). In humans, pathogenic mutations in *Mfn2* cause Charcot-Marie-Tooth type 2A (CMT2A) disease, characterized by progressive distal sensory and motor neuron abnormalities and distal muscular atrophy (Zuchner et al., 2004). Mutation of *Opa1* is associated with optic atrophy defects (Alexander et al., 2000; Delettre et al., 2000). *Drp1* knockout mice have severe neurological defects including abnormal brain development and neonatal lethality. This suggests that Drp1 is important for both embryogenesis and neurogenesis almost certainly due to its role in mitochondrial fission (Ishihara et al., 2009). A list of neurodegenerative diseases with mitochondrial phenotypes are listed in Table 1.

## Mitochondrial Dynamics and Transport

The close relationship between mitochondrial fusion/fission machinery and transport is noteworthy. Mitofusins (Mfn1 and Mfn2) physically interact with the RhoT/Trak complex. Inhibition of *Mfns* in cultured neurons and *in vivo* markedly reduce both the anterograde and retrograde transport (Misko et al., 2010). The fission protein Drp1 has also been implicated in mitochondrial transport. Inhibition of *Drp1* function disrupts mitochondrial transport to dendrites in Purkinje cells both *in vitro* and *in vivo* (Fukumitsu et al., 2016). In another study Drp1 was shown to be important for distribution of mitochondria in the nerve terminals of dopamine neurons (Berthet et al., 2014). Recent work has shown Drp1 modulates dynein-based retrograde transport through interaction with the dynein–dynactin complex (Drerup et al., 2017). Although we now know a great deal about the fusion and fission machineries, there are still outstanding questions that need to be addressed. Since fusion and fission are oftentimes both regulated by and directly influence other cellular organelles (endoplasmic reticulum, peroxisomes, etc.) as well as mitochondrial transport and function, it is still not clear if the pathological consequences of disrupted dynamics are causal in disease or merely a downstream consequence. With the advent of novel sensors, *in vivo* imaging platforms, and advanced microscopy techniques we will be able to separate each of these mitochondrial properties to gain insights into the mechanisms and function of mitochondrial dynamics.

## Concluding Remarks

Protein synthesis in neurons can occur both at the somatodendritic compartment and at the distal regions of axons and dendrites. Proteins synthesized in the soma need to be faithfully transported through the long length of neuron to the target region or organelle. Additionally, mRNAs can be transported with local translation occurring in distal neuronal compartments. Both mechanisms are utilized by mitochondria and evidence of local translation of mitochondrial transcripts in distal axon has reliably been shown. In either of the above-mentioned scenarios proper axonal transport of these components is essential for mitochondrial survival in neurons. Thus, intracellular transport machinery plays an important role in maintaining the mitochondrial pool along the extended neuronal arbor.

Although the majority of mitochondria are considered to be stationery in mature neurons, at least on time scales of minutes, in order to maintain a healthy pool of the organelle in distal regions, mitochondria rely on active transport. Evidence for this is shown in *Drosophila* motor axons where inhibition of Kinesin-1 stops mitochondrial movement in both direction and leads to a dramatic decrease in the density of the organelle (Pilling et al., 2006). Mutations of dynein or dynein accessory proteins can cause specific defects in the mitochondrial population moving toward the cell body (Pilling et al., 2006; Drerup et al., 2017). This implies that movement of components or the organelle itself is essential for both mitochondrial and neuronal health. Although transport defects have been associated with neurodegenerative disorders, to date no therapeutic interventions have been reported which includes manipulation of mitochondrial transport specifically; however, the possibility of mitochondria as a therapeutic target is of great interest. Defects in the localization, health, and function of this organelle are a commonality in numerous disease states (Reddy and Reddy, 2011). Thus, fundamental knowledge about mitochondrial transport dynamics *in vivo* may aid in the development of effective therapeutics for diseases involving transport defects of this organelle. Future *in vivo* studies with longer time frames will provide critical insight in to the transport dynamics along the entirety of a neuron.

## Author Contributions

AM did the literature search and wrote the initial draft. CD directed the manuscript preparation and edited the final version of the manuscript.

## Conflict of Interest Statement

The authors declare that the research was conducted in the absence of any commercial or financial relationships that could be construed as a potential conflict of interest.
